# Foreign RNA spike-ins enable accurate allele-specific expression analysis at scale

**DOI:** 10.1093/bioinformatics/btad254

**Published:** 2023-06-30

**Authors:** Asia Mendelevich, Saumya Gupta, Aleksei Pakharev, Athanasios Teodosiadis, Andrey A Mironov, Alexander A Gimelbrant

**Affiliations:** Altius Institute for Biomedical Sciences, 2211 Elliott Ave, Seattle, WA 98121, United States; Stem Cell Program, Boston Children’s Hospital, 300 Longwood Avenue, Boston, MA 02115, United States; Department of Stem Cell and Regenerative Biology, Harvard University, 7 Divinity Ave, Cambridge, MA 02138, United States; Altius Institute for Biomedical Sciences, 2211 Elliott Ave, Seattle, WA 98121, United States; Faculty of Bioengineering and Bioinformatics, Lomonosov Moscow State University, 1-73 Vorobiovy Gory, Lab. Bldg B, Moscow 119992, Russia; Institute of Information Transmission Problems, Russian Academy of Sciences, 19 Bolshoi Karetny per., Moscow 127994, Russia; Altius Institute for Biomedical Sciences, 2211 Elliott Ave, Seattle, WA 98121, United States

## Abstract

**Motivation:**

Analysis of allele-specific expression is strongly affected by the technical noise present in RNA-seq experiments. Previously, we showed that technical replicates can be used for precise estimates of this noise, and we provided a tool for correction of technical noise in allele-specific expression analysis. This approach is very accurate but costly due to the need for two or more replicates of each library. Here, we develop a spike-in approach which is highly accurate at only a small fraction of the cost.

**Results:**

We show that a distinct RNA added as a spike-in before library preparation reflects technical noise of the whole library and can be used in large batches of samples. We experimentally demonstrate the effectiveness of this approach using combinations of RNA from species distinguishable by alignment, namely, mouse, human, and *Caenorhabditis elegans*. Our new approach, controlFreq, enables highly accurate and computationally efficient analysis of allele-specific expression in (and between) arbitrarily large studies at an overall cost increase of ∼5%.

**Availability and implementation:**

Analysis pipeline for this approach is available at GitHub as R package controlFreq (github.com/gimelbrantlab/controlFreq).

## 1 Introduction

A highly informative approach to understanding gene regulation is allele-specific expression analysis in RNA-seq data from diploid organisms, including humans. The two parental alleles share the environment of the same nucleus, making allelic imbalance (AI) in gene expression reveal *cis*-regulation (Wittkopp and Kalay 2011; Nica and Dermitzakis 2013). AI analysis has been increasingly used for understanding regulatory variation (Pirinen et al. 2015; Moyerbrailean et al. 2016; GTEx_Consortium, 2017; Mohammadi et al. 2019; Uechi et al. 2020; Vierstra et al. 2020). Besides uncovering genetic effects, allele-specific analysis can reveal epigenetic gene regulation in *cis*, including imprinting (Tucci et al. 2019), X-chromosome inactivation (Galupa and Heard 2018), and autosomal monoallelic expression (Gimelbrant et al. 2007; Zwemer et al. 2012; Gendrel et al. 2014; Chess 2016; Vinogradova et al. 2019).

**Figure 1.**
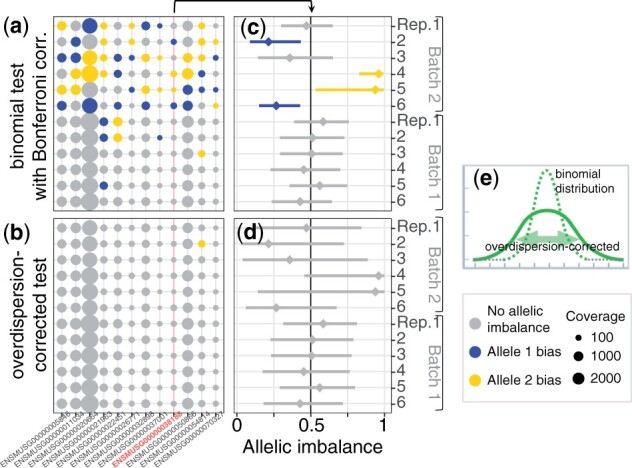
Allele-specific signal from RNA-seq can substantially vary dependent on technical noise. Analysis of replicates can be leveraged to account for this experiment-specific overdispersion. RNA-seq data: 12 technical replicates from the same RNA (129xCastF1 mouse kidney; from Mendelevich et al. (2021)). “Batch 1” and “Batch 2” were denoted Experiment 2 and Experiment 3 in that paper; both used the same library preparation kit (SMART-Seq v4 Ultra Low Input RNA Kit) with amounts of input total RNA bracketing recommended range: 10 ng per library for Batch 1, and 100 pg for Batch 2. (a) Shown are all genes (coverage >10 allelic counts) with conflicting assignment of allelic bias across 12 RNA-seq libraries from the same RNA. Gray—no AI, i.e. null hypothesis of AI = 0.5 cannot be rejected at *P* < 0.5 in the binomial test with Bonferroni correction. If null hypothesis rejected (i.e. gene is called as having AI): blue—significant prevalence of paternal allele; yellow—significant prevalence of maternal allele. Marker sizes reflect coverage (in total allelic counts); Allelic counts and statistical analysis performed using commonly used approach (e.g. GTEx_Consortium 2017); (b) Same data as in (a), with allelic counts table analysed using modified binomial test with overdispersion correction (AI overdispersion discussed in Mendelevich et al. (2021); (c–d) AI for one of the genes (ENSMUSG00000038188, mean coverage of 110 ± 45 allelic counts) denoted in red in (a–b). AI is defined as (maternal counts)/(sum of maternal and paternal counts), so that AI = 0 is maternal expression only, AI = 1 is paternal only, and AI = 0.5 is exactly biallelic. Diamond marker denotes the point estimate for this gene in each replicate library, which is by definition unchanged between (c) and (d). Confidence interval is determined by the statistical test used. Note overdispersion correction (b, d) has much more pronounced effect on confidence intervals in replicates from the more noisy datasets (Batch 2) compared to less noisy datasets (Batch 1); (e) Schematic representation of super-binomial overdispersion (solid line) as compared to binomial (dashed line).

In a previous work (Mendelevich et al. 2021), we have shown that substantial technical noise is present in RNA-seq experiments and significantly varies between experiments, but it is often not accounted for in the analysis. We demonstrated that in the absence of technical replicates, it is impossible to separate this technical noise from biological variation. The resulting underestimation of the technical noise leads to an increased number of false positives in AI calls and limited reproducibility between different studies (Fig. 1a). To quantify and account for this technical overdispersion (Fig. 1b), we have developed a statistical framework and software tool, Qllelic, that takes advantage of the production of two or more RNA-seq libraries for each RNA sample, i.e. technical replicates (Mendelevich et al. 2021). However, preparation and sequencing of replicate libraries at least doubles the experiment’s costs. This complicates practical application of this approach, especially for large projects, where cost of RNA-seq is a major component of the cost of the study.

Here, we describe an alternative approach for precise allele-specific analysis of large-scale RNA sequencing experiments involving only a small increase in costs. The experimental component of this approach consists of adding an aliquot of foreign, easily distinguishable RNA to every sample (e.g. RNA from heterozygous mouse to human RNA samples) as a spike-in before starting library construction.

Non-allelic RNA spike-in standards have been previously used to access technical noise in transcription levels measurements in bulk RNA-seq (Jiang et al. 2011; Lovén et al. 2012), and estimate background noise, technical batch effects, and doublets in scRNA-seq experiments (Brennecke et al. 2013; Grün et al. 2014; Kim et al. 2015). However, estimates of non-allelic abundance variation are of limited utility for allele-specific analysis (Mendelevich et al. 2021); thus, we aimed to develop an RNA spike-in approach that captures technical noise in AI measurements.

The analytical part of our approach relies on the uniformity of the spike-in RNA aliquots across all libraries. We extend our previously described idea of processing data from replicates to the spike-in portions of all the libraries in a batch. Crucially, we experimentally show that allele-specific noise properties of the spike-in portion reflect technical noise of the whole sample. Thus, spike-in acts as the standard for estimating technical noise in each library. Adding a fraction of spike-in RNA to the main sample of interest (e.g. mouse RNA to human RNA at 1:10 ratio) removes the need for additional libraries, while only marginally increasing total depth of sequencing, as opposed to doubling or tripling experimental costs.

## 2 Materials and methods

### 2.1 Measuring overdispersion using extended beta-binomial model

In this article, we revised and expanded the procedure described in Mendelevich et al. (2021). We recall that we define technical replicates as a set of separate libraries prepared from the same RNA, and thus capturing technical noise that accumulates starting from library preparation. Having several technical replicates, we can calculate pairwise Quality Correction Coefficients (QCCs), which one may consider as a widening coefficient of the expected binomial quantiles, for the replicates with similar library sizes. We made pairwise comparisons, and assigned an averaged overdispersion value to each replicate, considering QCC results for relevant pairs.

**Figure 2.**
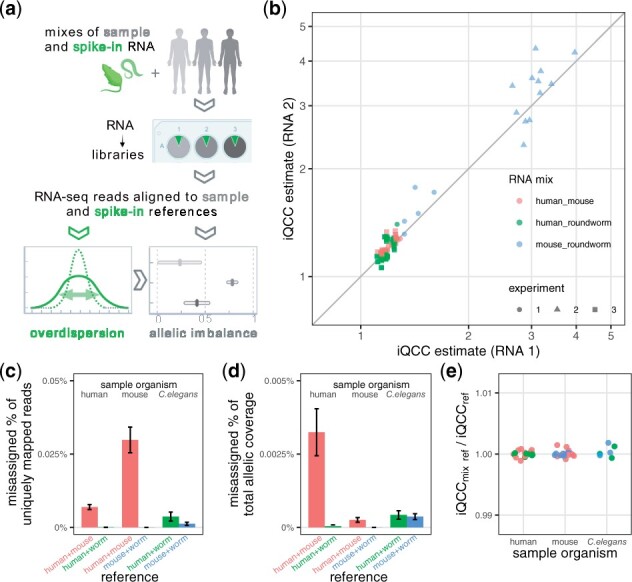
In libraries using mixes of RNA from two distinct organisms, both RNAs show similar allelic overdispersion. (a) Diagram of experimental and computational steps in AI estimation pipeline. Spike-in RNA (green) is added to the main sample (gray) prior to starting preparation of RNA-seq library. (b) Within an RNA-seq library, overdispersion statistic iQCC measured for the two distinct RNAs in a mix is highly similar (Pearson correlation 0.97). For description of libraries in Experiments 1-3, see Table 1. (c–e) Estimation of data loss due to cross-species alignment in data from mixed RNA-seq libraries. Data used: three human and three mouse biological replicates and one roundworm biological replicate (three technical replicates each), from Experiment 1, aligned on the individual or chimeric reference. Color represents reference mixture used in alignment, pair encoding is the same as for RNA mix in (b). (c) Percentage of misaligned reads to the wrong organism among all uniquely aligned reads. (d) Percentage of allelicaly-resolved counts aligned to the wrong reference. (e) Comparison of iQCC values calculated based on alignment to a single or to a mixed (chimeric) reference. (Note also that 0 genes with differential AI were found for each of possible pairs).

**Figure 3.**
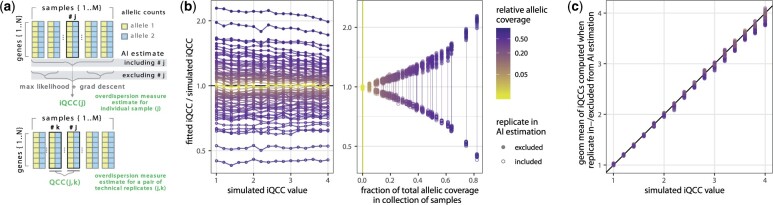
Calculation of iQCC is robust to the number of replicates (spike-ins) and to variation in library sizes within a batch. (a) Scheme of the iQCC calculation (using controlFreq) and previously described QCC calculation (using Qllelic) for a j-th sample in a batch. See Methods for details. (b) exclusion and inclusion of j-th replicate result in, respectively, underestimation and overestimation of iQCC from the total pool of replicates. Left panel—as allelic coverage increases, the upper and lower iQCCs estimates converge to ground truth (gold); right panel—extension of (a) shows symmetry for overestimated and underestimated measures (lines connect same replicate combinations). (c) same data as in (b), geometric means of underestimated and overestimated iQCC values clearly correlate with the generated levels of overdispersion. (b–c) Simulations of replicate data (all generative iQCCs were similar for all replicates, which mimics technical replication, or similar experimental environment) with different total coverage and overdispersion levels. For iQCC values between 1 (no overdispersion) and 4 (high overdispersion), gene allelic counts for 10 replicates (“libraries”) were generated using predetermined “ground truth” AI distribution and coefficients for total allelic coverage; the procedure was repeated thrice for every set of parameters. Upper and lower iQCC estimates were computed for each selected replicate.

Here we describe the new procedure, which operates with lots of samples at once (Fig. 2a) and assigns individual values of overdispersion measure, individual QCC (iQCC), to each sample in the batch (Fig. 3a). It might be applied in both scenarios: spike-ins use case and technical replication use case (see the respective subsections at the end of this section).

#### 2.1.1 Extended beta-binomial distribution

Beta-binomial family of distributions is commonly used to model an “overdispersed” binomial distribution. One way to parameterize this family is using the real parameters α≥0 and β≥0 which stand for the number of different balls in the Pólya urn model. Using this parameterization, the probability mass function of the beta-binomial distribution can be written as follows:


$${\text{pmf}}_{BB}(m|n=m+p,\alpha ,\beta )=\left(\begin{array}{c} n\\ m \end{array}\right)\frac{\prod_{i=0}^{m-1}\left(\alpha +i\right)\cdot \prod_{j=0}^{p-1}\left(\beta +j\right)}{\prod_{k=0}^{n-1}\left(\alpha +\beta +k\right)}.$$

The variance of this distribution is


$$\frac{\alpha +\beta +n}{\alpha +\beta +1}\cdot \frac{n\alpha \beta }{{\left(\alpha +\beta \right)}^{2}}.$$

The second part is the variance of the binomial distribution with $\text{AI}=\frac{\alpha }{\alpha +\beta }$, so we capture the overdispersion $\frac{\alpha +\beta +n}{\alpha +\beta +1}$ in a parameter we denote by *Q*. Given parameters 0≤AI≤1 and 1≤Q≤n, we can reconstruct back *α* and *β* as $\alpha =\text{AI}\frac{n-Q}{Q-1}$ and $\beta =(1-\text{AI})\frac{n-Q}{Q-1}$.

Two edge cases of the possibility range for *Q* are 1 and *n*. When *Q* approaches *n*, *α*, and *β* become close to 0, and the probabilities of all outcomes except for 0 and *n* tend to 0. The limit distribution assigns probability AI to 0, and probability AI to *n*. When *Q* approaches 1, *α* and *β* tend to +∞, and the distribution approaches the binomial distribution with the probability AI.

One drawback of the beta-binomial distribution family is that it does not allow us to model “underdispersed” distributions, in other words, the distributions with *Q* < 1. While most samples give overdispersed distributions, some could be modeled better with underdispersed ones (see Subsection 2.1.4 for more details), and clamping them to one point *Q* = 1 does not make computational sense. Fortunately, we can overcome the problem by extending the probability mass formulas past the edge *Q* = 1, essentially extrapolating the family of beta-binomial distributions into hypergeometric distributions following (Prentice 1986).

Consider the following modified Pólya urn model with the usual parameters *α* and *β* and an additional parameter *d*. Place α≥0 balls of the first type and β≥0 balls of the second type into the urn. Draw *n* balls from the urn as usual. The modification is how we replenish the urn. In the beta-binomial case, after drawing a ball we put two balls of the same type in the urn, making the total number of balls in the urn larger by 1 on each step. In our modified case, we put *d* + 1 balls instead of 2, increasing the total by *d* each time. The probability mass function of this extended beta-binomial distribution is quite similar to the usual one, except that the increment in the multipliers is *d* instead of 1:


$$\begin{array}{c} {\text{pmf}}_{\mathrm{eBB}}(m|n=m+p,\alpha ,\beta ,d)=\\ =\left(\begin{array}{c} n\\ m \end{array}\right)\frac{\prod_{i=0}^{m-1}\left(\alpha +id\right)\cdot \prod_{j=0}^{p-1}\left(\beta +jd\right)}{\prod_{k=0}^{n-1}\left(\alpha +\beta +kd\right)}. \end{array}$$

We can see a few properties of this definition right away.

1. The extended distribution does not depend on the scaling of the parameters, i.e. for any *x* > 0
  $$\begin{array}{c} {\text{pmf}}_{\mathrm{eBB}}(m|n=m+p,\alpha x,\beta x,dx)=\\ ={\text{pmf}}_{\mathrm{eBB}}(m|n=m+p,\alpha ,\beta ,d). \end{array}$$
2. If *d* = 1, the extended distribution coincides with the beta-binomial distribution with the parameters *α* and *β*.
3. If *d* = 0, the extended distribution coincides with the binomial distribution with $\text{AI}=\frac{\alpha }{\alpha +\beta }$.
4. A hypergeometric distribution ${\text{pmf}}_{HG}(m|n=m+p,M,P)$ belongs to this family as
  $${\text{pmf}}_{\mathrm{eBB}}(m|n=m+p,\alpha =M,\beta =N,d=-1).$$

Therefore, this continuous family of distributions contains all beta-binomial, binomial, and hypergeometric distributions. From the property 1 we gather that the family is 2D rather than 3D, and can be described using the parameters 0≤AI≤1 and Q≤n just by analytically continuing the description of the beta-binomial distribution. The reparametrization using *AI* and *Q* can be put in the following neat form:


$$\begin{array}{c} {\text{pmf}}_{\mathrm{eBB}}(m|n=m+p,\text{AI},Q)=\\ ={\text{pmf}}_{\mathrm{eBB}}\left(m|n=m+p,\alpha =\text{AI},\beta =1-\text{AI},d=\frac{Q-1}{n-Q}\right). \end{array}$$

The binomial distribution with *Q* = 1 stops being an edge case, since the range *Q* < 1 becomes treatable, and now we can model underdispersed distributions alongside with the overdispersed ones.

#### 2.1.2 Finding Q which maximizes likelihood

For the purpose of using this distribution efficiently in our models, we implement fast and accurate calculation of the density function ${\text{pmf}}_{\mathrm{eBB}}(m|n,\text{AI},Q)$ as follows.

The defining formula of the density function of the extended beta-binomial distribution is not suitable for the numerical application right away. Both the numerator and the denominator are orders of magnitude larger than the actual value of the expression, so a straightforward implementation would lose precision. To avoid that, we match each multiplier in the numerator with a suitable multiplier in the denominator, calculate their ratio, and only then take the product. Also, since we are usually interested in the log-likelihood function, we employ one more trick to increase the accuracy of the calculations. Instead of calculating the logarithm of each fraction directly, we calculate it using the function log(1+x). In R language, this function is called log1p. Overall, we have


$$\begin{array}{l} \;\text{log}\;{\text{pmf}}_{\mathrm{eBB}}(m|n,\alpha ,\beta ,d)=\\ =-\sum_{i=0}^{m-1}\mathrm{log1p}\left(\frac{\beta }{\alpha +\beta +id}\right)+\\ +\sum_{j=0}^{p-1}\;\mathrm{log1p}\left(\frac{-(j+1)\alpha +m\beta -md}{\left(j+1)(\alpha +\beta +(m+j)d\right)}\right). \end{array}$$

The differential of this expression is straightforward.

These calculations constitute a major building block in the optimization problem which is at the heart of the top-level fitting procedures.


$$\begin{array}{l} \mathrm{Given}\;a\;\text{vector}\;\text{of}\;\text{allele}\;\text{counts}\;m_{l},p_{l},0\leq l\leq g-1,\\ \text{and}\;a\;\text{vector}\;\text{of}\;\text{corresponding}\;\text{AI}\;\text{estimations}\;{\text{AI}}_{l},0\leq l\leq g-1,\\ \mathrm{Find}\;Q\;\text{which}\;\text{maximizes}\;\text{the}\;\text{likelihood}\;\text{function}\\ L(Q)=\prod_{l=0}^{g-1}{\text{pmf}}_{\mathrm{eBB}}(m_{l}|m_{l}+p_{l},{\text{AI}}_{l},Q). \end{array}$$

We tackle the problem using the gradient ascent on the logarithm of the likelihood function. The calculation of $\frac{d\;\text{log}\;L(Q)}{dQ}$ is straightforward and can be easily translated into code. The rest of the implementation comes down to the “flavours” of the gradient ascent.

#### 2.1.3 Computing overdispersion with spike-ins

One of the most important features of the spike-in protocol is a large number of samples involved in overdispersion calculation. This implies that the total allelic counts in spike-in batch are expected to be much higher than in each of the samples. Modeling of the allelic counts in this case is challenging, since in general the sum of two beta-binomial random variables does not need to be beta-binomial. To simplify, we make an assumption that the overdispersion coefficients are approximately the same, in which case the sum will still be beta-binomial, so $m_{1}+m_{2}∼\mathrm{eBB}(n_{1}+n_{2},\text{AI},Q={\text{iQCC}}^{2})$ when $Q_{1}≃Q_{2}$, *m_i_* stand for the maternal counts, and *n_i_* stand for the total counts. Spike-in samples do not meet technical replication criteria, but they share RNA source. Then the best estimate of AI might be computed from the whole spike-in batch: for a selected sample *j* and a set of ground truth $Q_{i\neq j}$ values. This estimate can be considered coming from a beta-binomial distribution with *N* being the total coverage of the other samples. When *N* is of several orders of magnitude larger than *n*, the estimate of ${\text{AI}}_{j}$ does have negligible variance, and the procedure for fitting the best $Q_{j}={\text{iQCC}}_{j}^{2}$ will work well. However, when *n* and *N* are of the same order or *N* is less than *n*, the estimate may deviate from the real ${\text{AI}}_{j}$, and the value of *Q_j_* will inevitably be overestimated. This can be particularly challenging if the amount of samples is low. But usually in the spike-in case we have N≫n. See Fig. 3a, b for how much *Q_j_* will be overestimated depending on the ratio n/(n+N).


(1) $${\text{AI}}_{j}=\frac{\sum_{i\neq j}\frac{m_{i}}{Q_{i}}}{\sum_{i\neq j}\frac{\left(m_{i}+p_{i}\right)}{Q_{i}}}$$

If all of *Q_i_* values in a set of samples or N≫n are of similar order, it should be enough to run the fitting algorithm once. The first run of the algorithm sets all *Q_i_* to the same value (*Q_i_* = 1). Thus Equation (1) simplifies to ${\text{AI}}_{j}=\sum_{i\neq j}m_{i}/\sum_{i\neq j}\left(m_{i}+p_{i}\right)$.

Otherwise, if *Q_i_* estimates from the first run show significant dispersion, the fitting algorithm can be iterated. In the grand scheme, we alternate between fitting *Q*’s, then fitting AI’s, and so on. The AI fitting is run using the formula (1), and the *Q* fitting is run using the gradient ascend algorithm. After each *Q*-fitting step, *Q_i_* values are compared to previous estimates, and iterations are repeated until convergence.

#### 2.1.4 Computing overdispersion in case of technical replication

In case of small number of samples, we need to take care of the overestimation of *Q_j_*. To do that, we counter the previous estimate with a different one. If one includes the sample *j* in the ${\text{AI}}_{j}$ estimation, then the corresponding fitted *Q_j_* will be underestimated. Moreover, on the simulated data we see that the overestimated and underestimated iQCC values deviate from the real value by the same amount in the logarithmic scale, see Fig. 3b. We use this fact in the case of technical replication, where we fit ${\text{iQCC}}_{j}$ to be the geometrical mean of the overestimate and the underestimate to get a more precise statistic (Fig. 3c;Supplementary Fig. S2). Using this more precise fitting, we can safely use the pipeline on a small amounts of samples, given a prior knowledge that the samples have similar overdispersion.

### 2.2 Data

Information on RNA-seq datasets generated for this work, including sources of RNA, library preparation methods, sequencing, and data processing is summarized in Table 1.

**Table 1.** Description of the data used in this work

EXPT #	1			2		3		
GEO SubSeries	GSE228002			GSE228003		GSE228004		
Samples	**18 libraries** with mixes of human (H), mouse (M) and *C. elegans* (W) RNA. H1: M1, H1: W and M1: W (75:25 and 50:50 of total allelic coverage), x3 tech. replicates each. W RNA is a 1:1 mix of N2 and Haw RNA. **21 libraries** with single-species RNA: (H1, H2, H3, M1, M2, M3, W) x 3 technical replicates each. H1-3 and M1-3 are biological replicates (cells from separate wells).			**12 libraries**: mixes of RNA from 3 separate mice with *C. elegans* spike-ins W1, W2 and W3 (N2: Haw RNA mixes of 1:1, 2:1, 1:2, respectively). **Including**: 6 libraries M1 (spleen+W1, spleen+W2, liver 1+W1, liver 1+W2, liver 2+W1, liver 2+W3); 4 libraries M2 (spleen+W1, spleen+W2, liver+W1, liver+W2); 2 libraries M3 (liver+W1, liver+W3).		**32 libraries**: Lymphocyte RNA from 3 donors (H1-H3), with M and W (1:1 N2: Haw) spike-ins. Including: set of (H1a, H1b, H2, H3) x (M RNA 10% of total, M 25%, M 50%)—12 libraries total; (H1a, H1b, H2, H3) x (W 10%, W 25%, W 50%)—12 libraries; (H1a, H1b, H2, H3) x 2 replicates w/out spike-ins—8 libraries. **9 libraries**: ((H4, H5, H6, H7) + M 10%) x 2 biological replicates, H1c + M 10%; **9 libraries—**same with W 10%; **9 libraries** of N2: Haw RNA mixes (1:1, 3:1, 1:3) x3 technical replicates each.		
Data	Illumina 150PE			Illumina 150PE		Illumina 151PE		
Lib prep kit	NEBNext Single Cell/Low Input RNA			NEBNext Single Cell/Low Input RNA		SMART-Seq v4 PLUS		
Organism	Human	Mouse	*C. elegans*	Mouse	*C. elegans*	Human	Mouse	*C. elegans*
RNA prep^a^	Agilent Mini	Agilent Mini	Trizol	Trizol	Trizol	Qiagen Mini	Agilent Mini	Trizol
Genotypes^b^	NA12878	129xCastF1 (Abl.1 clone)	RNA mix (N2 + Hawaii)	129xCastF1 (organs, bio replicates)	RNA mix (N2 + Hawaii)	Donors 1–7^c^	129xCastF1 (Abl.1 clone)	RNA mix (N2 + Hawaii)
References	GRCh38.p13	GRCm38.68	PRJNA13758.WS276	GRCm38.68	PRJNA13758.WS276	GRCh38.p13	GRCm38.68	PRJNA13758.WS276
Variants	Illumina Pt genomes, 2017	Sanger dbSNP142, 2014	CeNDR, soft-filtered, 2020	Sanger dbSNP142, 2014	CeNDR, soft-filtered, 2020	Imputed, this study	Sanger dbSNP142, 2014	CeNDR, soft-filtered, 2020

a RNA prep: Agilent Absolutely RNA miniprep (Agilent Mini), Qiagen RNeasy miniprep (Qiagen Mini).

b Genotypes: 129S1/SvImJ x CAST/EiJ F1 (129XCastF1).

c Of donors: #1 present in 3 bio replicates, #2-5—in 2, #6-7—in 1; M1, M2, and M3 and W1, W2, and W3 notations are different for different experiments, see descriptions.

#### 2.2.1 Biological material

v-Abl pro-B clonal cell lines Abl.1 derived previously from 129S1/SvImJ × Cast/EiJ F1 female mice (Zwemer et al. 2012) and human clonal pro-B cell line GM12878.DF1 (Nag et al. 2013) were cultured in RPMI medium (Gibco), containing 15% FBS (Sigma), 1X L-Glutamine (Gibco), 1X Penicillin/Streptomycin (Gibco) and 0.1% *β*-mercaptoethanol (Sigma). Human peripheral blood monocytes from deidentified donors were appropriately consented for generating genomic sequencing data for unrestricted deposition in public databases. *Caenorhabditis elegans* strains N2 and CB4856 (Hawaiian) were maintained at 20°C on nematode growth medium plates with Escherichia coli OP50 bacteria (Brenner 1974).

#### 2.2.2 RNA preparation

Biological replicates for human and mouse clones (see Table 1) were made by growing cells in separate wells of a 6-well plate at a seeding density of 500 000 per milliliter (1 500 000 total cell per well). RNA isolation and DNase treatment for both clonal cell lines was performed using Absolutely RNA Microprep Kit (Agilent) according to instruction. RNA from *C. elegans* was prepared using Trizol reagent (Invitrogen), with DNase treatment using TURBO DNA-free kit (Ambion). RNA integrity was assessed using Bioanalyzer and was quantified using Qubit RNA HS Assay. DNase-treated RNA from N2 and Hawaiian strains was mixed in proportions described in Table 1. For RNA preparation from mouse tissues, whole tissues were collected from adult 129xCastF1 mice housed at the DFCI mouse facility, with parent animals obtained from the Jackson Laboratories. All animal work was performed under DFCI protocol 09-065, approved by the DFCI Institutional Animal Care and Use Committee. Animals were housed in accordance with Guide for the Care and Use of Laboratory Animals. Collected tissues were crushed using mortar and pestle in liquid nitrogen. This powdered tissue was either taken directly for RNA isolation using Trizol reagent or stored in liquid nitrogen for later use.

#### 2.2.3 Library preparation and sequencing

For experiments 1 and 2 (see Table 1), aliquots of total RNA were used to prepare libraries using NEBNext Single Cell/Low Input RNA Library Prep Kit (NEB). The libraries were sequenced at Novogene on the Illumina NovaSeq platform and 150 bp paired-end reads were generated. For experiment 3 (see Table 1), libraries were prepared using SMART-Seq v4 PLUS kit (Takara), and PE-151 sequencing was performed on Illumina NovaSeq at the High Scale Data Unit at Altius Instutite for Biomedical Sciences.

#### 2.2.4 Variant calling and imputation

Imputation on genotype calls for donor samples in experiment #3 was performed on autosomes only. VCFTools was used to split files per chromosome. Per chromosome genotype files were submitted to the TopMed imputation server (imputation.biodatacatalyst.nhlbi.nih.gov) with the following parameters: imputation = minimac4-1.0.2; phasing = eagle-2.4; panel = TOPMED.vR2; Mode: Quality Control & Imputation. About 131 562 sites were excluded prior to imputation, leaving 572 955 sites for imputation. About 131 452 of the excluded sites were due to monomorphic sites. After imputation, only markers with a R2 value of ˃9 were kept. *De novo* SNPs were annotated with dbSNP v151.

### 2.3 Generation of allelic count tables

#### 2.3.1 Reference preparation

To receive *in silico* chimeric pseudogenome reference files “containing” more than one organism, we first create a set of respective pseudogenome reference files for each of them, namely, pseudo-reference fasta files for both alleles (reference genome with inserted SNPs related to one fixed haplotyped allele), allelic VCF files (SNPs only, and 1st and 2nd alleles playing role of reference and alternative respectively) and reference GTF files. The procedure is described in detail in Mendelevich et al. (2021). Next, we merge them with a consistent renaming of similar chromosome names via appending the distinguishing suffixes (e.g. chromosome 1 may be renamed to “1 m” in mouse and “1 h” in human). These resulting files are used as “reference” files in all the next steps. Finally we index an *in silico* chimeric pseudogenome with STAR, setting sjdbOverhang parameter to the advised data-specific len(read)-1.

#### 2.3.2 Data pre-processing

Note that we have revised recommendations on allelic counts tables creation relative to Qllelic, to minimize effect of noise coming from statistically dependent counts for nearby variants while preprocessing RNA-seq data (see GitHub for details: github.com/gimelbrantlab/controlFreq). The unit of overdispersion analysis is a table with allelic read counts per gene. In order to obtain that kind of tables, we first align the reads to both pseudogenomes with STAR (version 2.7.9a), which supports allele-aware alignment (–outSAMattributes vA vG) when provided prearranged reference fasta and vcf (see section above for details). For further analysis, we select reads aligned at positions that include at least one SNP to determine the allele, and filter out those whose SNP signals or alignment positioning on two haplotypes don’t show consistency. The remaining reads are assigned to one of the alleles, according to SNP calls and alignment quality. Finally, we assign allele-resolved reads to genes and calculate gene counts using featureCount (version 2.0.2) (with parameters –countReadPairs -B -C). Autosomal genes were used for iQCC values calculation.

## 3 Results

### 3.1 Mixes of RNA from highly distinct organisms show similar overdispersion in all components

**Figure 4.**
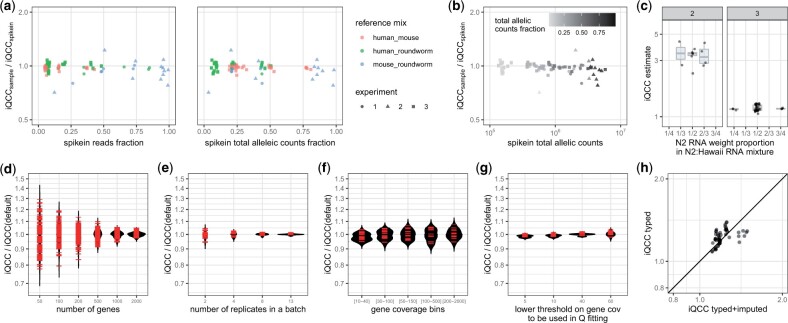
Overdispersion estimate is robust to wide range of variation in the spike-in quantity and composition. (a) Ratio of iQCC values for sub-library from organism 1 and from organism 2 (assigned as “main sample” and spike-in) remains close to 1 with diminishing fraction of spike-in RNA in the initial mix (see Table 1 for details); (b) Ratio of iQCC values for organism 1 and organism 2 for different total allelic counts (reflecting library size) and the spike-in fraction. Note decreased fidelity for extremely low spike-in counts (tens of thousands allelic reads). Note: in the case of equal mix compounds organism 2 is assigned to “spike-in sample”; (c) Polymorphism in the spike-in RNA can be mimicked by a mix of RNA from two distinct strains, with no strict requirement that mix is exactly 1:1. iQCC values are robust across mixes of RNA from N2 and Haw strains of *C. elegans* with different allelic ratios; (d) iQCC computed on randomly sampled N genes for 13 replicates (10% spike-in samples), shown for each replicate separately. Each gene sampling was performed 10×; (e) iQCC computed on different collections of samples: 11 pairs, 3 quadruples, 3 eights and 2 batches of 13 replicates; (f) iQCC computed in different allelic coverage bins, on 13 replicates (10% spike-in samples), only genes whose coverage belong to the interval in all 13 replicates were considered (number of genes per bins: 611, 610, 399, 496, and 232); (g) iQCC computed with different coverage threshold for fitting, using whole data, on 13 replicates (10% spike-in samples); (d–g) Data: experiment 3, mouse spike-ins. Default iQCC values are computed with fitting threshold on coverage = 30 and in a batch of all 21 spike-in samples, without any other restrictions; (h) iQCC computed on experiment #3 samples containing human RNA, when only typed or typed and imputed variants are considered.

We showed previously (Mendelevich et al. 2021) that AI overdispersion in poly-A RNA-seq experiments behaves like a consistent multiplicative parameter from binomial expectations, across all genes in a sample. We hypothesized that if we mix RNA from two highly distinct species, the overdispersion will be similar for all components. We developed the experimental and computational protocol (operating with evolved overdispersion measure iQCC, individual QCC, see Fig. 2a), and have shown the proximity of overdispersion measured across different regions of the genome, including chromosomes of different species origins in our *in-silico* chimeras (see Figs 2b and 4; Supplementary Fig. S2).

Notably, human, mouse and roundworm data can be mapped simultaneously without too much interference. The number of uniquely mapped reads changed by hundredths of a percent (Supplementary Fig. S3) for mapping human samples to the mouse-human reference, while ˂0.03% of uniquely mapped reads are crossed-mapped (see Fig. 2c–e). There were no genes showing differential allelic imbalance (AI) when comparing mapping to combined reference and mapping to single-species reference.

### 3.2 Use of one RNA across many samples can serve as a “stand-in” for tech replicates

Overdispersion measurement analysis on spike-in components differs from analysis of technical replicates, since here we should not be bound by the strict assumption of shared overdispersion between all samples present in a batch. Aiming to meet the changed criteria we had to modify and improve our previous overdispersion calculation pipeline, which currently takes advantage either from same overdispersion (technical replicates case) or from replicates quantity (spike-in case), and utilizes the assumption of shared initial state of allelic proportions in RNA prep in both scenarios (see Fig. 3a, and Methods). Finally, overdispersion estimates (iQCC) obtained with the current procedure are highly correlated with Qllelic results (see Supplementary Fig. S1).

### 3.3 Spike-in protocol is flexible and allows variation of parameters

Aiming to determine the limitations of spike-in methodology, we varied and tested different conditions. Combinations of different organisms pairs, mixed in different proportions, resulted in comparable overdispersion measurements on both components (see Fig. 4a). More specific, the only limitation is total allelic coverage (see Fig. 4b, f), which clearly depends on the spike-in proportion, total number of sequenced reads and SNP density in an organism (for human it is on the order of 1/1000, for mice and roundworm samples we used it is 1/100 and 1/500, respectively). Moreover, mixes of RNA prepared from individual parental strains of *C. elegans* (N2 and Hawaiian (CB4856)) performed as a spike-in just as well as N2xHawaiian F1 heterozygous cross (see Figs 2b and 4a). We also showed that N2 and Hawaiian RNA could be mixed in a range of proportions with similar overdispersion estimates (see Fig. 4c and Discussion). This makes experimental preparation of spike-ins much easier than worm or mouse husbandry.

## 4 Discussion

The spike-in approach we describe here, including the data-processing pipeline, is much more accurate and precise in estimating allele-specific expression than the common single-replicate design (Fig. 1). In fact, it is at least as accurate as Qllelic, the approach with technical replicates for each sample we described previously, but rather than costing several-fold higher, the cost increases only by ∼5–10%.

As previously discussed (Mendelevich et al. 2021), allele-specific analysis of an RNA-seq experiment without technical replicates is subject to a fundamental uncertainty regarding the contribution of technical noise to false positive rate. This risk is emphasized by the magnitude of technical noise that can be encountered. For example, iQCC > 4 observed in Fig.  2b is equivalent to overestimating allelic coverage over 16-fold by the binomial test. Thus, common thresholding techniques (such as “at least 10 reads”) can be dangerously misleading.

The new approach, controlFreq, supersedes our previous one, Qllelic, and incorporates its functionality.

The improvements of computation protocol include moving from pairwise overdispersion measure QCC, computed by Qllelic on pairs of technical replicates, to batch-wide calculation of iQCC for each sample.

When controlFreq is used to analyse experiments with a small number of replicates (e.g. 2 or 3), it has the same limitation as Qllelic, namely, the assumption of overdispersion similarity between replicates, even as it no longer requires similarity in library sizes. However, when multiple replicates are available (as spike-ins), the new approach enables confident detection of outliers, allows us to remove the assumption of similarity of overdispersion in all samples, and eliminates expectations of library size similarity. Overall, the analysis is highly robust to fluctuations.

An important practical feature of the spike-in approach is that the spike-in RNA must be identical only within the batch of samples being analysed. Once the overdispersion corrections are calculated for the batch of samples, they can be correctly compared to samples from any other batch, including those using entirely different spike-ins (or, indeed, any sample with properly estimated AI overdispersion). This means that spike-in RNA can be prepared as needed, and does not have to come from a single source. It should be possible to use other spike-ins in parallel. For example, existing designs using sequins (Hardwick et al. 2016) or ERCC for cell number control (Munro et al. 2014), could be used if desired, without interference with the AI spike-ins described here.

As far as we can establish, the only requirements of the spike-in material are (i) that it has significant density of polymorphisms (the higher the better) and (ii) its nature enables it to undergo the same library preparation process as the main sample. So, e.g. bacterial RNA should not be used for poly-A-based libraries, or *C. elegans* RNA as a spike-in standard for mammal samples when using library protocol with ribo-depletion. Otherwise, spike-in material can be produced in any convenient way; we posit that once the “snapshot” of allelic proportions is captured for the whole library, the origin of library components does not matter. For example, we used “synthetic F1s”—mixes of RNA from homozygous *C. elegans* strains (see Fig. 4c) as a spike-in, which is easier than breeding F1 crosses. It might be possible to use yeast RNA as a spike-in, or develop a set of synthetic molecules for allele-resolved RNA-seq analysis, analogous to ERCC controls (Jiang et al. 2011). Notably, only a very modest coverage is needed for the spike-in: as few as 125 000 allelic counts were sufficient for effective use of spike-ins (see Fig. 4a, b). One consequence is that a spike-in with high SNP density requires few total reads. As we discussed previously (Mendelevich et al. 2021), the overdispersion correction appears equally applicable across the genome.

The allelic counts depend on read length, both in terms of total sequence data and unique alignment rate: e.g. with 50PE reads, total allelic counts are about 1/4 of the counts with 150PE reads (Supplementary Fig. S3). The desired coverage of the main sample depends on the study aims. A useful guide is that to reliably detect 80:20 imbalance in a gene (accounting for Bonferroni correction for analysis with 1000 genes), the sufficient allelic count coverage is roughly $50\times ({\text{iQCC}}^{2})$; $110\times ({\text{iQCC}}^{2})$ for 70:30 imbalance, and $420\times ({\text{iQCC}}^{2})$ for 60:40 imbalance.

We note that the experiments we described are focused on poly-A-enriched bulk RNA sequencing. While we expect that application of this approach can be extended into a variety of other experimental settings where accurate measurement of allelic signal is desirable, a pilot study should be performed in each particular case to test the applicability of spike-in methodology and tune it accordingly. A clear example of an application is capturing technical noise in single-cell RNA-seq; this is a question of great importance since otherwise (Kim et al. 2015), no technical replication is readily available at the single-cell level (attempts to split single-cell RNA into two replicates are extremely technically challenging (Deng et al. 2014)). It is known that the rate of overdispersion in single-cell analysis is high, underscoring the need for reliable methods for distinguishing technical noise from meaningful biological variation. Finally, a similar approach might be applicable to other types of libraries besides RNA, such as ChIP-seq, ATAC-seq, and analysis of DNA methylation.

## Supplementary Material

btad254_Supplementary_Data

## Data Availability

Sequencing data is deposited in GEO as record GSE228009 (https://www.ncbi.nlm.nih.gov/geo/query/acc.cgi?acc=GSE228009). The subseries GSE228002, GSE228003 and GSE228004 correspond to experments 1, 2, and 3.
